# Factors associated with the incidence and the expenditure of self-medication among middle-aged and older adults in China: A cross-sectional study

**DOI:** 10.3389/fpubh.2023.1120101

**Published:** 2023-04-13

**Authors:** Yuxin Liu, Zehao Zheng, Xiubo Wang, Jiabei Xia, Xingce Zhu, Fanjun Cheng, Zhiyong Liu

**Affiliations:** ^1^School of Medicine and Health Management, Tongji Medical College, Huazhong University of Science and Technology, Wuhan, China; ^2^School of Pharmacy, Tongji Medical College, Huazhong University of Science and Technology, Wuhan, China; ^3^Institute of Hematology, Union Hospital, Tongji Medical College, Huazhong University of Science and Technology, Wuhan, China

**Keywords:** self-medication, pharmaceutical expenditure, older people, China, two-part model

## Abstract

**Background:**

With the accelerated ageing of population and the growing prevalence of various chronic diseases in China, self-medication plays an increasingly important role in complementing the health care system due to its convenience and economy.

**Objective:**

This study aimed to investigate the incidence of self-medication and the amount of self-medication expenditure among middle-aged and older adults in China, and to explore factors associated with them.

**Methods:**

A total of 10,841 respondents aged 45 years and older from the China Health and Retirement Longitudinal Study (CHARLS) wave 4 which conducted in 2018 were included as the sample of this study. The two-part model was adopted to identify the association between the incidence of self-medication and the amount of self-medication expenditure and specific factors, respectively.

**Results:**

The incidence of self-medication among Chinese middle-aged and older adults was 62.30%, and the average total and out-of-pocket (OOP) pharmaceutical expenditure of self-medication of the self-medicated individuals were 290.50 and 264.38 Chinese yuan (CNY) respectively. Participants who took traditional Chinese medicine (TCM), self-reported fair, and poor health status, suffered from one and multiple chronic diseases had strongly higher incidence of self-medication. Older age and multiple chronic diseases were strongly associated with higher expenditure of self-medication. Those who took TCM had more self-medication expenditure, while those who drank alcohol had less.

**Conclusion:**

Our study demonstrated the great prevalence of self-medication among middle-aged and older adults in China and the large pharmaceutical expenditure that come with it, especially in the high-risk groups of self-medication identified in this paper. These findings enhanced our understanding of self-medication behaviors among Chinese middle-aged and older adults and may contribute to the formulation of targeted public health policy.

## Introduction

Self-medication is defined as patient’s personal choice to use medication to treat self-perceived illness or symptom without professional guidance (1), including a wide range of conditions from the common cold to various chronic diseases (2). Forms of self-medication include the use of over-the-counter medication, leftover medication from a previous prescription, medication delivered by friends or relatives, and so on (3). When facing illness, it is a universal practice worldwide for patients to alleviate their symptoms through self-medication, especially for middle-aged and older people who experiencing minor ailments (2). A systematic review showed that the majority of studies reported the incidence of self-medication among older adults varied between 20 and 60% (4). The prevalence of self-medication among middle-aged and older people is acknowledged as a public health issue and been discussed all over the world.

The popularity of self-medication is inseparable from its convenience, rapidity, and affordability (5). For patients, self-medication reduces treatment costs and saves consultation time (3, 6). For the government, self-medication reduces the burden of limited medical service resources by making better use of physicians and pharmacists’ skills (7, 8). Thus, responsible self-medication avoids unnecessary medical treatment and medical consultation, providing significant economic benefits for both individuals and health care system (9, 10). However, on the other hand, due to the lack of professional guidance, the negative effects of inappropriate self-medication cannot be ignored, including drug abuse, drug side effects, and drug interactions, which may cause harm to patients’ health (11). It is worth noting that considering their vulnerability to multimorbidity (12), older people are prone to greater drug consumption and higher risk of inappropriate drug use. Furthermore, inappropriate use of medications would also lead to more serious adverse drug reactions in older adults due to age-related changes in the pharmacodynamics and pharmacokinetics of medications (13). In addition, although self-medication is economical compared with hospital-based health care methods such as inpatient and outpatient treatment, it has also been found to incur significant costs due to inadequate insurance coverage (14). All of the above mentioned jointly raise concerns about self-medication behavior, especially among middle-aged and older people.

Several previous studies have attempted to identify potential determinants of self-medication behaviors. Figueiras et al. found that self-medication was significantly associated with gender, living conditions, and place of residence among respondents in Spain (15). A study of European countries by Grigoryan et al. revealed that the level of wealth had a negative impact on the possibility of residents’ self-medication (16). As for studies conducted on middle-aged and older Chinese population, Wang et al. found that participants who with medical insurance, living in non-rural areas and western region were more likely to take self-medication (1). Besides, there are also some studies have attempted to explore the factors influencing pharmaceutical expenditure, particularly out-of-pocket (OOP) pharmaceutical expenditure. Park et al. found that in Korea, due to more chronic conditions and worse economic status, older adults had greater OOP pharmaceutical expenditure (17). The study by Look and Arora revealed that OOP pharmaceutical expenditure of prescription medications decreased by 30% after the expansion of health insurance coverage among American adults (18), indicating that health insurance also affects pharmaceutical expenditure to a large extent. Study on Chinese population by Du et al. found that health insurance, poor self-reported health status, and chronic or critical diseases significantly increased the amount of OOP pharmaceutical expenditure of self-medication (19). However, existing relevant studies in Chinese context only considered the type of medical insurance, the association between insurance utilization and self-medication pharmaceutical expenditure has not been discussed. In addition, the role played by traditional Chinese medicine (TCM) also deserves attention. In China, TCM was considered to be an important complement to western medicine for treating chronic or serious diseases, and was widely used to guide self-care practices among Chinese patients (20). Although previously conducted studies generally classified the use of TCM as self-medication behavior (21), the existing studies have insufficiently discussed Chinese patients’ self-medication behaviors from a TCM perspective, and the relevance of TCM and self-medication remains unclear. Therefore, there is a need to understand the self-medication behavior of Chinese middle-aged and older adults from a more comprehensive perspective based on the latest data.

Our study focuses on the self-medication of middle-aged and older adults in China, who are generally considered to be relatively unhealthy and economically disadvantaged in China. This age group has more health care needs, tends to choose self-medication and is also more vulnerable to the risks of it (22–24). The purpose of this study is to investigate the incidence of self-medication and the amount of self-medication expenditure among middle-aged and older adults in China, to explore factors associated with them and to develop targeted opinions. The findings of this study will contribute to the comprehensive understanding of the determinants of self-medication behaviors among middle-aged and older Chinese population, and provide reference information for the formulation of related public health policy to promote responsible self-medication, curb inappropriate self-medication and alleviate self-medication expenditure.

## Methods

### Data and sample

This study used data from the China Health and Retirement Longitudinal Study (CHARLS) which targeting middle-aged and older adults in China. CHARLS is a nationally representative longitudinal survey of Chinese residents aged 45 years and older conducted by the National School of Development of Peking University (25). The survey of CHARLS included people from 450 villages or communities in 150 counties in 28 provinces of China using multi-stage stratified probability-proportionate-to-size sampling, representing the whole country to a large extent (26). The questionnaire of CHARLS covers a wide range of information, including demographic background, family, health status, health care, insurance, income, and so on, which can meet the study needs on the health of middle-aged and older people. All CHARLS data were collected through computer-assisted personal interviews, using a structured questionnaire. And all the CHARLS respondents provided written informed consent before the data were collected. Both data and questionnaire were available on the corresponding website (27).

**Figure 1:**
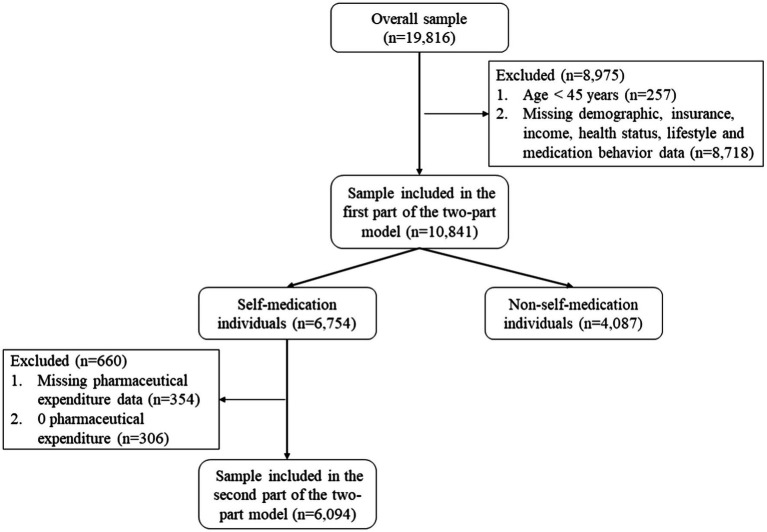
Flow chart of the detailed process of enrolling participants in the two-part model.

This study used data from wave 4 of CHARLS conducted in 2018, which collected information from roughly 19,000 respondents (25). Middle-aged and older adults aged 45 years and older who provided complete information on all relevant variables were included in the first part of the two-part model. The exclusion criteria were: (1) age < 45 years; (2) missing demographic, insurance, health status, lifestyle, and medication behavior data. As a result, a total of 10,841 respondents were included in the analysis of the first part of the two-part model. On this basis, the sample of the second part of the two-part model was further narrowed to participants who provided complete information on pharmaceutical expenditure. In addition, respondents who reported 0 pharmaceutical expenditure were also excluded to make the analysis feasible. The exclusion criteria were: (1) missing pharmaceutical expenditure data; (2) 0 pharmaceutical expenditure. Ultimately, a total of 6,094 participants were included in the analysis of the second part of the two-part model. The sampling process is shown in detail in Figure 1.

### Ethical approval

Ethics approval for the data collection in CHARLS was obtained from the Institutional Review Board (IRB) at Peking University (IRB00001052-11015).

### Measures

#### Dependent variables

Measures of self-medication was based on the question “Did you take any purchased medicine during the past month (Not including prescription medications)? Taking any medicine delivered by others or stored by oneself is also counted.” Thus, a binary variable of self-medication was created. And for respondents who reported having self-medication behaviors, the amount of pharmaceutical expenditure of self-medication was measured by the question “What is the approximate total cost for purchased medicine during the last month (Include out-of-pocket part and reimbursement part)?” while the amount of OOP pharmaceutical expenditure of self-medication was measured by the question “How much did you pay out-of-pocket?”

#### Independent variables

The independent variables were selected based on the Andersen health service utilization model (28). This model summarized the factors affecting the utilization of personal health services into three dimensions, namely predisposing, enabling, and need factors. Predisposing factors included demographics and health beliefs. Enabling resources included socioeconomic and insurance. Need factors were related to personal perceived or diagnosed health care needs. Since its creation in the late 1960s, this model has been widely used to explain the utilization patterns of health care services (29).

In this study, age, gender, marital status, education level, and place of residence were considered as predisposing factors. In addition, the common use of TCM among middle-aged and older adults in China was also taken into account and included as predisposing factor. For enabling factors, we included individual income level and health insurance status. Besides, insurance utilization containing union reimbursement was also included in the analysis of self-medication expenditure. As to need factors, self-reported health status, number of chronic diseases, drinking, and smoking status were incorporated.

Specifically, age was divided into two categories: 45–59 and 60 years and older. Gender was categorized into male and female. Marital status was categorized into married or not. Education was classified into three levels: no formal education, elementary school, and below and middle school and above (27). Residence was categorized into rural and urban areas. Use of TCM was categorized into having or not. Income was classified into three levels: low defined as 10,000 Chinese yuan (CNY) and less, moderate defined as 10,001 CNY to 50,000 CNY and high defined as more than 50,000 CNY, and 1 United States Dollar (USD) = approx. 6.5 CNY. Health insurance was divided into five statuses: no health insurance, urban employee medical insurance (UEMI), urban and rural resident medical insurance (URRMI), urban resident medical insurance (URMI), and new rural cooperative medical insurance (NRCMI) (19). Insurance utilization was divided into having or not. Self-reported health was classified into three levels: good, fair, and poor. Number of chronic diseases was divided into three categories: no chronic diseases, one chronic disease, and multiple chronic diseases. Drinking was categorized into having or not in the past year. Smoking was categorized into currently having or not (23).

### Statistical analysis

In the univariate analysis, descriptive analysis was conducted to compare respondents’ characteristics by having self-medication or not. Corresponding Chi-square values and values of *p* were estimated by Pearson’s Chi-squared tests. Then, in the multivariate analysis, similar to most current studies on medical expenditure based on the CHARLS database, our study adopted the two-part model for data analysis (30). The two-part model is divided into two parts for the measurement of continuous data such as medical expenditure. The first part focuses on the probability of receiving health treatment, exploring the factors affecting the acceptance of health treatment; The second part focuses on the medical expenditure, estimating the individuals with positive medical expenditure to analyze the effect of factors on medical expenditure. Specific to the practical application of this study, in the first part, binary logistic regression was used to analyze the factors associated with the probability of self-medication behavior, corresponding odds ratios (OR), and 95% confidence interval (CI) of each factor were reported. In the second part, factors associated with the total and OOP pharmaceutical expenditure amounts of self-medication were analyzed separately using generalized linear models adopting a log link with Gamma distribution, corresponding regression coefficient and significance level of each factor were reported. Analyses were conducted using Stata 15.0.

## Results

### Characteristics of the overall sample

The descriptive statistics of all the participants were presented in Table 1. A total of 6,754 respondents reported having self-medication behaviors during the past month, accounting for 62.30% of the whole sample, indicating that self-medication is predominant among Chinese middle-aged and older adults. In the overall sample, there were about a quarter of middle-aged people aged 45–59 and about three quarters of older people aged 60 and above, and there were slightly more women than men. The majority of participants were concentrated in the low-income group, with only a small proportion located in the high-income group, and there were more participants lived in rural areas than those who lived in urban areas. Most of the participants were married, educated, and insured. The majority of participants reported not good health status, and about half of the participants had at least one chronic disease. There were fewer participants took TCM, and participants who smoked or drank were also in the minority. In the univariate analysis, age, gender, income level, self-reported health, and chronic diseases status, having TCM, drinking and smoking or not were seen significantly associated with the probability of taking self-medication. Older participants, female participants, participants with low income, poor health status, and multiple chronic diseases, participants who having TCM, not drinking and not smoking were more likely to administer self-medication.

**Table 1:** Basic information of all the respondents in the survey (*n* = 10,841).

Characteristics	Total	Self-medication		χ^2^	*p* value
		Yes (*n* = 6,754)	No (*n* = 4,087)		
Age					
45–59	3,020 (27.86)	1,773 (26.25)	1,247 (30.51)	22.995	0.000
≥60	7,821 (72.14)	4,981 (73.75)	2,840 (69.49)		
Gender					
Male	5,406 (49.87)	3,265 (48.34)	2,141 (52.39)	16.656	0.000
Female	5,435 (50.13)	3,489 (51.66)	1,946 (47.61)		
Marital status					
Married	9,042 (83.41)	5,624 (83.27)	3,418 (83.63)	0.241	0.624
Single	1,799 (16.59)	1,130 (16.73)	669 (16.37)		
Education					
No formal education	2,462 (22.71)	1,555 (23.02)	907 (22.19)	2.128	0.345
Elementary school and below	4,699 (43.34)	2,939 (43.52)	1,760 (43.06)		
Middle school and above	3,680 (33.95)	2,260 (33.46)	1,420 (34.75)		
Residence					
Rural	7,528 (69.44)	4,698 (69.56)	2,830 (69.24)	0.119	0.730
Urban	3,313 (30.56)	2,056 (30.44)	1,257 (30.76)		
Having traditional Chinese medicine					
Yes	3,152 (29.07)	2,342 (34.68)	810 (19.82)	272.540	0.000
No	7,689 (70.93)	4,412 (65.32)	3,277 (80.18)		
Income					
Low	6,457 (59.56)	4,156 (61.53)	2,301 (56.30)	30.071	0.000
Moderate	3,777 (34.84)	2,250 (33.32)	1,527 (37.36)		
High	607 (5.60)	348 (5.15)	259 (6.34)		
Insurance status					
Uninsured	260 (2.40)	158 (2.34)	102 (2.50)	3.837	0.429
UEMI	2,089 (19.27)	1,270 (18.80)	819 (20.04)		
URRMI	1,337 (12.33)	837 (12.39)	500 (12.23)		
URMI	491 (4.53)	318 (4.71)	173 (4.23)		
NRCMI	6,664 (61.47)	4,171 (61.76)	2,493 (61.00)		
Self-reported health					
Good	2,215 (20.43)	1,048 (15.52)	1,167 (28.55)	375.453	0.000
Fair	5,463 (50.39)	3,387 (50.15)	2,076 (50.80)		
Poor	3,163 (29.18)	2,319 (34.33)	844 (20.65)		
Chronic diseases					
No chronic diseases	5,347 (49.32)	3,081 (45.62)	2,266 (55.44)	126.439	0.000
One chronic disease	3,371 (31.10)	2,161 (31.99)	1,210 (29.61)		
Multiple chronic diseases	2,123 (19.58)	1,512 (22.39)	611 (14.95)		
Drinking					
Yes	3,805 (35.10)	2,277 (33.71)	1,528 (37.39)	15.084	0.000
No	7,036 (64.90)	4,477 (66.29)	2,559 (62.61)		
Smoking					
Yes	2,965 (27.35)	1,761 (26.07)	1,204 (29.46)	14.690	0.000
No	7,876 (72.65)	4,993 (73.93)	2,883 (70.54)		

### Binary logistic regression on self-medication incidence

In the first part of the two-part model on the possibility of self-medication, binary logistic regression was used to explore the factors associated with the incidence of self-medication, and the reference group of the dependent variable was “having no self-medication behavior.” Corresponding OR, 95% CI and significance level of each independent variable were presented in Table 2. In the analysis, the adoption of self-medication was found strongly associated with having TCM, self-reported health, and chronic diseases status. Specifically, participants who took TCM (OR = 1.816; *p* < 0.001) were more likely to have self-medication behaviors. Compared participants who reported good health, those who reported fair (OR = 1.693; *p* < 0.001) and poor health (OR = 2.495; *p* < 0.001) tend to take self-medication. Self-medication behaviors were also more likely to occur in participants with one (OR = 1.179; *p* < 0.001) and multiple (OR = 1.400; *p* < 0.001) chronic diseases than in those without chronic conditions. Besides, female (OR = 1.115; *p* = 0.040) was found positively associated with the possibility of self-medication. With the no-education group as the baseline, participants with middle school education and above (OR = 1.189; *p* = 0.011) were found significantly higher probability of self-medication. And for income level, based on the low-income group, individuals with moderate income (OR = 0.876; *p* = 0.016) were less likely to take self-medication at a significance level.

**Table 2:** Binary logistic regression on self-medication incidence (*n* = 10,841).

Characteristics	OR	95% CI	*p* value
Age (ref: 45–59)			
≥60	1.072	0.970–1.185	0.173
Gender (ref: Male)			
Female	1.115	1.005–1.236	0.040
Marital status (ref: Married)			
Single	0.912	0.815–1.020	0.106
Education (ref: No formal education)			
Elementary school and below	1.073	0.960–1.200	0.212
Middle school and above	1.189	1.040–1.359	0.011
Residence (ref: Urban)			
Rural	0.972	0.868–1.088	0.617
Having traditional Chinese medicine (ref: No)			
Yes	1.816	1.652–1.997	0.000
Income (ref: Low)			
Moderate	0.876	0.786–0.976	0.016
High	0.879	0.714–1.083	0.227
Insurance status (ref: Uninsured)			
UEMI	1.105	0.827–1.476	0.501
URRMI	1.159	0.874–1.537	0.305
URMI	1.194	0.858–1.663	0.294
NRCMI	1.071	0.823–1.393	0.610
Self-reported health (ref: Good)			
Fair	1.693	1.529–1.875	0.000
Poor	2.495	2.208–2.820	0.000
Chronic diseases (ref: No chronic diseases)			
One chronic disease	1.179	1.076–1.292	0.000
Multiple chronic diseases	1.400	1.250–1.569	0.000
Drinking (ref: No)			
Yes	1.012	0.922–1.112	0.795
Smoking (ref: No)			
Yes	0.948	0.855–1.052	0.316

### Pharmaceutical expenditure among the self-medicated sample

In the analysis conducted on the expenditure of self-medication, insurance utilization containing union reimbursement was also taken into account. After excluding respondents who did not report self-medication behavior and reported 0 self-medication expenditure, a total of 6,094 participants were included in the analysis, which were presented in Table 3.

**Table 3:** Total and OOP pharmaceutical expenditure amounts of self-medication in CNY among the self-medicated respondents (*n* = 6,094).

Characteristics	Total pharmaceutical expenditure		OOP pharmaceutical expenditure	
	Mean	SD	Mean	SD
Age				
45–59	239.00	585.55	216.07	463.90
≥60	309.35	865.59	282.07	797.43
Gender				
Male	296.63	723.17	261.01	546.31
Female	284.67	868.07	267.58	859.08
Marital status				
Married	291.58	843.41	263.31	757.81
Single	285.01	535.45	269.82	519.09
Education				
No formal education	283.77	1132.76	269.46	1128.32
Elementary school and below	261.83	626.40	237.76	479.62
Middle school and above	333.70	727.78	296.70	632.30
Residence				
Rural	255.73	817.52	236.23	749.63
Urban	374.35	752.47	332.30	652.69
Having traditional Chinese medicine				
Yes	394.90	818.49	362.85	678.51
No	234.03	785.34	211.13	741.86
Income				
Low	273.56	864.68	254.94	794.54
Moderate	309.51	659.09	274.13	554.41
High	389.93	800.14	326.08	769.32
Insurance status				
Uninsured	287.08	458.51	280.09	454.78
UEMI	449.97	812.05	385.46	734.89
URRMI	239.46	382.99	222.92	361.92
URMI	346.06	916.44	305.27	646.70
NRCMI	255.58	852.13	237.82	782.36
Self-reported health				
Good	225.12	1374.98	206.44	1363.18
Fair	240.55	639.79	212.68	452.09
Poor	390.02	648.10	363.22	625.87
Chronic diseases				
No chronic diseases	245.92	955.37	221.34	864.03
One chronic disease	284.50	609.16	259.99	537.00
Multiple chronic diseases	389.27	682.01	357.77	631.27
Drinking				
Yes	238.36	524.18	215.28	412.89
No	316.81	907.88	289.17	837.02
Smoking				
Yes	246.40	542.73	223.84	430.15
No	306.10	873.56	278.73	802.00
Insurance utilization				
Yes	322.24	610.78	224.29	363.12
No	281.51	846.68	275.73	796.49
Total	290.50	800.82	264.38	723.89

1 USD = approx. 6.5 CNY.

Overall, the average total and OOP pharmaceutical expenditure of self-medication of the 6,094 self-medicated individuals were 290.50 and 264.38 CNY, respectively. Specific to each factor, both total and OOP pharmaceutical expenditure of self-medication were seen to increase with age and income level, but did not differ significantly by gender or marital status. Compared with uneducated participants, individuals with elementary school education and below had less self-medication expenditure, while individuals with middle school education and above had more. There were both higher total and OOP pharmaceutical expenditure of self-medication for participants living in urban areas. It is also worth noting that individuals who took TCM had significantly higher self-medication expenditure than those who did not. The expenditure of self-medication was also seen rising with participants’ worse health status and increased number of chronic conditions. Participants who maintained smoking and drinking habits were found to spend less on self-medication. In terms of health insurance status, participants enrolled in UEMI paid the highest self-medication expenditure while those enrolled in URRMI paid the lowest. In addition, individuals who used insurance had higher total pharmaceutical expenditure of self-medication but lower OOP pharmaceutical expenditure of self-medication compared to those who did not use insurance.

### Generalized linear model evaluation of self-medication expenditure

In the second part of the two-part model on the expenditure of self-medication, generalized linear model adopting a log link with Gamma distribution was used to identify variables significantly associated with the total and OOP pharmaceutical expenditure amounts of self-medication separately. Corresponding regression coefficient and significance level of each independent variable were presented in Table 4. In the analysis, both total and OOP pharmaceutical expenditure of self-medication were found significantly associated with age, residence, having TCM, and drinking. Specifically, older participants aged 60 years and above were found to have a higher amount of self-medication expenditure compared with middle-aged participants aged 45–59 years. Participants living in urban areas had higher self-medication expenditure than those living in rural areas. Individuals who took TCM had more self-medication expenditure, while those who kept drinking habits had less. In addition, the health status of the participants was also found to be associated with the self-medication expenditure. Compared with respondents reported good health, those who reported poor health had higher self-medication expenditure. And compared with individuals without chronic diseases, the self-medication expenditure for individuals with multiple chronic conditions was higher. It should also be noted that participants who used insurance (estimated coefficient = −0.199; *p* = 0.001) had lower OOP pharmaceutical expenditure of self-medication than those who did not, but no significant difference was found in the total pharmaceutical expenditure of self-medication.

**Table 4:** Generalized linear model evaluation of the total and OOP pharmaceutical expenditure amounts of self-medication in CNY (*n* = 6,094).

Characteristics	Total pharmaceutical expenditure		OOP pharmaceutical expenditure	
	Coefficient	*p* value	Coefficient	*p* value
Age (ref: 45–59)				
≥60	0.215	0.001	0.207	0.001
Gender (ref: Male)				
Female	−0.109	0.169	−0.039	0.535
Marital status (ref: Married)				
Single	−0.122	0.142	−0.095	0.234
Education (ref: No formal education)				
Elementary school and below	−0.160	0.160	−0.166	0.131
Middle school and above	0.034	0.778	0.042	0.726
Residence (ref: Urban)				
Rural	−0.148	0.018	−0.143	0.018
Having traditional Chinese medicine (ref: No)				
Yes	0.393	0.000	0.389	0.000
Income (ref: Low)				
Moderate	−0.071	0.289	−0.077	0.229
High	0.038	0.788	0.026	0.858
Insurance status (ref: Uninsured)				
UEMI	0.200	0.336	0.179	0.388
URRMI	−0.308	0.119	−0.230	0.243
URMI	0.021	0.941	−0.077	0.749
NRCMI	−0.254	0.210	−0.219	0.275
Self-reported health (ref: Good)				
Fair	0.030	0.877	−0.004	0.984
Poor	0.428	0.032	0.454	0.023
Chronic diseases (ref: No chronic diseases)				
One chronic disease	0.028	0.704	0.037	0.596
Multiple chronic diseases	0.258	0.001	0.276	0.000
Drinking (ref: No)				
Yes	−0.249	0.000	−0.215	0.000
Smoking (ref: No)				
Yes	−0.154	0.052	−0.114	0.095
Insurance utilization (ref: No)				
Yes	0.127	0.061	−0.199	0.001

## Discussion

With the accelerated ageing of population and the increasing prevalence of various chronic diseases among middle-aged and older people, the demand for health care utilization in China also increases rapidly. Due to financial difficulties and inconvenient access to medical care, self-medication is an economical and convenient option for treating minor ailments, especially for middle-aged and older adults. As an important complement to hospital-based health care methods, the role of self-medication should not be ignored. Our study provided a comprehensive description of participants’ self-medication behaviors, attempted to explore the factors associated with the incidence of self-medication and the amount of self-medication expenditure among middle-aged and older adults in China. The findings of this study help to improve the understanding of self-medication behaviors of Chinese middle-aged and older adults, and contribute to the formulation of targeted public health policy.

The results of this study showed that 62.30% of the whole 10,841 Chinese middle-aged and older samples reported self-medication during the past month, which was slightly higher than previous study conducted on similar population. And after excluding the participants with missing data and those who reported 0 pharmaceutical expenditure, the average total and OOP pharmaceutical expenditure of self-medication of a total of 6,094 self-medicated individuals were 290.50 CNY and 264.38 CNY respectively, which were also higher than previously conducted study (19). The results indicated an increasingly trend of self-medication among middle-aged and older adults in China and the growing pharmaceutical expenditure that come with it. According to prior study, the popularity of self-medication in this group can be attributed to its cost and time savings (31).

Our findings suggested that the need factors of the Andersen health service utilization model are important factors driving the adoption of self-medication among middle-aged and older adults in China. Participants’ health status, including self-reported health and number of chronic diseases, was found to be strongly associated with the use of self-medication. Self-medication behaviors were more likely to occur in participants who reported fair and poor health or those with one and multiple chronic diseases, which was consistent with the findings of previously conducted studies on similar population, that is, poor health status forced them to use more health services primarily in the form of self-medication to recover from diseases (19, 23). The benefits of taking medicines are the original motivation for participants to take medicines, including the control of diseases and the improvement of the quality of lives (32), playing a direct role in promoting self-medication in middle-aged and older Chinese population. Another factor found to be strongly associated with taking self-medication was using TCM. Our results showed that participants who took TCM were more likely to take self-medication compared to those who did not. It may be explained by the Chinese patients’ preference for TCM (20), which result in the prevalence of self-medication with TCM among middle-aged and older people in China. In addition, there were several other factors found to be associated with the use of self-medication, including gender, education and income. Specifically, female population was more likely to engage in self-medication behaviors, which was consistent with the results of prior studies (33). Taking the no-education group as the baseline, the probability of self-medication was significantly higher among participants with middle school education and above. Similar preferences for health self-care among those with higher education level were also found in previous studies (23). Highly educated individuals had greater knowledge about diseases and medicines, as well as better cognitive capacity and health awareness (34, 35). In addition, education also improved self-efficacy to make self-diagnosis and self-treatment decisions (23). Therefore, high education level may increase the possibility of self-medication. As to income, based on the low-income group, those with moderate income were found less likely to take self-medication at a significance level. Similar negative correlation between income level and self-medication behavior was found in previous study as well (19). This may be due to the better economic conditions of the relatively wealthy enable them to choose adequate treatment such as visiting medical practitioner instead of taking self-medication (23, 36).

In the second part of the two-part model, several factors significantly associated with self-medication use were also found to be significantly associated with self-medication expenditure, such as the use of TCM and health status including self-reported health and number of chronic diseases. In addition to the above-mentioned preference of Chinese middle-aged and older people on taking TCM for self-medication, another important reason that increases the self-medication expenditure of individuals having TCM may be that TCM is often used as supplementary treatment, which is relatively expensive and difficult to be reimbursed (2). Also mentioned above, due to the benefits of taking medicines, such as controlling diseases and improving the quality of lives (32), self-medication is more likely to occur in individuals with bad health conditions. Therefore, higher self-medication expenditure is more likely to incur in this population as well. Specifically, individuals with multiple chronic diseases or reported poor health had significantly higher self-medication expenditure, which was consistent with the trend found in previous study (19). In addition, drinking is another strong predictor of the self-medication expenditure, suggesting that the need factors of the Andersen health service utilization model are not only important driving factors of self-medication adoption in Chinese middle-aged and older adults, but also have a profound impact on the expenditure of their self-medication behaviors. However, no significant association was found between smoking and the self-medication expenditure. An empirical study founded that self-medication with alcohol for heart and vascular diseases, insomnia, and emotional disorders was common in older adults (37). This may explain the negative association between drinking and self-medication expenditure, that is, middle-aged and older people consume alcohol instead of medicines for self-treatment. Another factor strongly associated with the expenditure of self-medication was age, older people aged 60 and above were found to spend more on self-medication than middle-aged people aged 45–59. This can be partly explained by the worse health status of older adults, who were prone to multiple chronic diseases and therefore require greater medication expenditure. On the other hand, as individual’s age and their disease burden increase, their perceptions of the value of medication may also convert with the reduction of their remaining lives, making them change their consumption patterns and exhaust their life savings on medications (38). The last factor significantly associated with both total and OOP pharmaceutical expenditure of self-medication was residence. Individuals living in urban areas had higher self-medication expenditure than those living in rural areas. Large urban–rural medical consumption gap in China was also reflected by previous study (39), which can be attributed to the income disparity between urban and rural areas in China (40). Furthermore, easier access to pharmacies in urban areas may also have contributed to greater self-medication expenditure (1, 41). In addition, our study found a strong association between insurance utilization and lower OOP pharmaceutical expenditure of self-medication, which may suggest that the use of insurance played a direct role in reducing the OOP pharmaceutical expenditure of self-medication. However, no significant association was found between the insurance utilization and the total self-medication pharmaceutical expenditure. Besides, it should also be noted that health insurance status was not found to be significantly associated with both the incidence of self-medication and the self-medication expenditure, which can be ascribed to the limited benefit packages provided by health insurance in China (19), such as covering fewer self-purchased medicines (13). Since the required medicines were not covered by health insurance, in many cases middle-aged and older people had to bear most or all of the pharmaceutical expenditure themselves, indicating that health insurance coverage has not significantly alleviated their burden of self-medication expenditure in China (19).

The findings of this study may contribute to government policymaking. Firstly, our study found that the phenomenon of self-medication was widespread among middle-aged and older adults in China. Considering its great prevalence, it is necessary for the government to incorporate self-medication into monitoring framework. In particular, the supervision of prescription drug sales in pharmacies needs to be strengthened to ensure the quality of pharmacy services. Secondly, the identification of high-risk groups of self-medication in this paper will help the government to develop targeted public health policy. Specifically, more attention should be paid to those who were found to take more self-medication and pay more for self-medication in this study, such as those taking TCM and suffering from chronic diseases. Policy intervention is needed to enhance health education for these vulnerable populations, especially in the area of rational drug use, in order to improve their responsible self-medication behaviors. In addition, policy support in the form of financial subsidies is also required to alleviate their OOP pharmaceutical expenditure of self-medication. Thirdly, it was also found in our study that health insurance coverage had no significant impact on self-medication behavior, suggesting that the government should make efforts to strengthen the role of health insurance. Consideration could be given to increasing welfare allowances and expanding benefit packages to protect individuals from large self-medication expenditure, thereby relieving the disease burden of middle-aged and older people.

## Limitations

It is worth noting that our study has certain limitations. Firstly, in CHARLS, respondents filled out the questionnaire based on their own memory, so there may be some recall and response errors in the collected data, especially in the aspect of pharmaceutical expenditure, leading to biased results in this study. However, given the fact that self-medication behaviors cannot be supervised and recorded by reliable institutions, the use of the questionnaire to collect information in CHARLS may be the most appropriate way. Secondly, considering its cross-sectional design, our study cannot provide evidence of causality. The analyses performed in this study can only indicate statistical correlations rather than causal relationships between self-medication behaviors and specific factors among Chinese middle-aged and older adults. However, CHARLS used a different questionnaire from the previous one to collect information on participants’ self-medication behaviors in the latest conducted survey, which may cause data deviation between the previous and subsequent waves of the survey. Therefore, it may not be appropriate to design a longitudinal study on self-medication using the latest CHARLS data. Thirdly, since all analyses conducted in this study used data from CHARLS, caution is needed when comparing the results of this study with those of other studies that use other questionnaires to measure self-medication behaviors. Although CHARLS represents middle-aged and older people in China to a large extent, it should still be cautious when generalizing our findings to other settings, especially to other countries. Nevertheless, we believe that the explanation of the relationship between self-medication behaviors and specific factors among Chinese middle-aged and older adults in this paper can still provide reference value for other studies.

## Conclusion

Our study focused on the comprehensive description of self-medication among Chinese middle-aged and older adults. In this study, self-medication was found prevalent among middle-aged and older adults in China and resulted in considerable pharmaceutical expenditure. Based on the Andersen health service utilization model, several factors were selected to be included in the analysis of this study, and some of which were found to be strongly associated with self-medication behaviors of Chinese middle-aged and older adults. Specifically, taking traditional Chinese medicine, self-reported fair and poor health status, having one and multiple chronic diseases were found strongly associated with higher probability of taking self-medication. As to self-medication expenditure, individuals who were older and had multiple chronic diseases had greater self-medication expenditure. Those who took traditional Chinese medicine had higher self-medication expenditure, while those who drank alcohol had lower. Insurance utilization was found strongly associated with less OOP pharmaceutical expenditure of self-medication. The findings of this study, especially the identification of the above-mentioned risk factors for self-medication provides clues for the formulation of targeted public health policy to regulate self-medication behaviors of middle-aged and older adults in China.

## Data availability statement

The datasets analyzed in this study are publicly available at the China Health and Retirement Longitudinal Study (CHARLS) site: https://charls.charlsdata.com/pages/Data/2018-charls-wave4/en.html.

## Ethics statement

The studies involving human participants were reviewed and approved by the Institutional Review Board (IRB) at Peking University (IRB00001052-11015). The patients/participants provided their written informed consent to participate in this study.

## Author contributions

YL developed the research design, performed the data analysis, and drafted the manuscript. ZZ helped the data analyses. XW and JX contributed the interpretation of results. XZ and FC revised the manuscript. ZL developed the research design and approved the final draft. All authors contributed to the article and approved the submitted version.

## Funding

This study was funded by the National Key Research and Development Plan of China (grant number 2020YFC2006000) and the National Natural Science Foundation of China (grant number 71874059). The funders had no role in study design, data collection and analysis, interpretation of data, and writing of the manuscript or decision to publish the paper.

## Conflict of interest

The authors declare that the research was conducted in the absence of any commercial or financial relationships that could be construed as a potential conflict of interest.

## Publisher’s note

All claims expressed in this article are solely those of the authors and do not necessarily represent those of their affiliated organizations, or those of the publisher, the editors and the reviewers. Any product that may be evaluated in this article, or claim that may be made by its manufacturer, is not guaranteed or endorsed by the publisher.

## Abbreviations

OOP: Out-of-pocket
TCM: Traditional Chinese medicine
CHARLS: China health and retirement longitudinal study
UEMI: Urban employee medical insurance
URRMI: Urban and rural resident medical insurance
URMI: Urban resident medical insurance
NRCMI: New rural cooperative medical insurance
OR: Odds ratios
CI: Confidence interval
